# First-admissions in Psychiatry: a cluster analysis of a sample of inpatients from 2019 to 2021

**DOI:** 10.1192/j.eurpsy.2023.1902

**Published:** 2023-07-19

**Authors:** E. La Spina, M. Mastrangelo, B. Montalbani, I. Mancinelli, A. Del Casale, A. Comparelli, M. Pompili

**Affiliations:** 1 Psychiatry Residency Training Program, Faculty of Medicine and Psychology, Sapienza University of Rome; 2 Department of Psychiatry, Sant’Andrea Hospital of Rome; 3 Department of Neurosciences, Mental Health and Sensory Organs, Faculty of Medicine and Psychology, Sant’Andrea Hospital, Sapienza University of Rome, Rome, Italy

## Abstract

**Introduction:**

Only a few studies analyse the clinical and psychopathological characteristics of first-admitted inpatients regardless of diagnosis.

**Objectives:**

Describing the psychopathological, demographic, and clinical characteristics of inpatients with acute symptomatology identifying groups with common features using factors extracted from the Brief Psychiatric Rating Scale (BPRS).

**Methods:**

We selected 103 (48 F) inpatients from the psychiatric ward of the Sant’Andrea Hospital in Rome from January 2019 to December 2021. We assessed psychopathological characteristics and suicide risk with BPRS, Global Assessment of Functioning, and Columbia-Suicide Severity Rating Scale and gathered the anamnestic and demographic data. We conducted descriptive analyses and factor analysis on BPRS items. Then we used the BPRS factors as variables to perform a cluster analysis.

**Results:**

Major Depressive Disorder (MDD) was the most frequent diagnosis. We obtained five factors: “Psychotic dimensions” (FI); “Anxiety” (FII); “Hostility and lack of cooperation” (FIII); “Depression” (FIV); “Flattening of affectivity” (FV). We identified two clusters (cluster 1 n=31; cluster 2 n=72). Patients in cluster 1 reported higher average scores in FI and FIII while the average scores of cluster 2 patients in FII and FIV were higher than patients in cluster 1. We called cluster 1 “psychotic and hostile patients compulsory admitted with a low risk of suicide”. Cluster 2 patients are “affective patients with a high risk of suicide”. The two clusters share an average age of 38-39 yo and an average GAF score indicating severe impairment and inability to function in almost all areas. They differ in the psychiatric diagnosis represented: respectively, Schizophrenia Spectrum Disorder and Bipolar Disorder with low suicidal risk, MDD, and Personality Disorders with a high suicidal risk. 39% of patients in cluster 1 were involuntarily admitted.

**Conclusions:**

The results of our study show that patients admitted for the first time usually are admitted for psychotic symptoms and a high risk of suicide. Psychotic patients more often show hostility and lack of cooperativeness which can explain the higher rate of involuntary admissions. Patients with predominant affective symptoms show a higher risk of suicide. Our analyses do not consider categorical diagnosis highlighting that exist transdiagnostic groups of patients with specific needs.

**Disclosure of Interest:**

None Declared

